# A study of time-fractional model for atmospheric internal waves with Caputo-Fabrizio derivative

**DOI:** 10.1371/journal.pone.0302743

**Published:** 2024-07-31

**Authors:** Miguel Vivas-Cortez, Maasoomah Sadaf, Zahida Perveen, Ghazala Akram, Sharmeen Fatima

**Affiliations:** 1 Escuela de Ciencias Físicas y Matemáticas, Facultad de Ciencias Exactas y Naturales, Pontificia Universidad Católica del Ecuador, Apartado, Quito, Ecuador; 2 Department of Mathematics, University of the Punjab, Quaid-e-Azam Campus, Lahore, Pakistan; 3 Department of Mathematics, Lahore Garrison University, Lahore, Pakistan; University of Education, PAKISTAN

## Abstract

The internal atmospheric waves are gravity waves and occur in the inner part of the fluid system. In this study, a time-fractional model for internal atmospheric waves is investigated with the Caputo-Fabrizio time-fractional differential operator. The analytical solution of the considered model is retrieved by the Elzaki Adomian decomposition method. The variation in the solution is examined for increasing order of the fractional parameter *α* through numerical and graphical simulations. The accuracy of the obtained results is established by comparing the obtained solution of considered fractional model with the results available in the literature.

## 1 Introduction

Internal waves are gravity waves which arise inside a fluid medium, in place of fluid surface. The internal waves are not easily observed from above the surface, but their observation and comprehension is essential in the study of layered fluid structures. Internal waves may arise when a tidal flow pushes the layered water body over a shallow underwater obstacle, like a sill or a ridge. In a similar fashion, the atmospheric internal waves may arise when air strikes a large obstacle which disturb the horizontal layers of homogeneous air. The study of atmospheric internal waves is significant to understand the occurrence of thunderstorms, rapid local temperature and pressure changes and clear air turbulence [1–3].

The atmospheric internal waves may be described by a system of shallow-water equations. A variety of studies have been conducted by many researchers on the internal waves phenomenon. Liggest and Woolhiser [4] solved shallow-water equation by employing finite difference methods. Flyer and Wright [5] analyzed shallow-water equations on the sphere by using radial basis function method. Turkington *et al*. [6] analyzed solitary internal waves in a continuously stratified fluid using a computational method. Delis and Katsaounis [7] presented the application of relaxation methods for numerical solution of two-dimensional shallow water equation. Aguilar and Sutherland [1] studied internal waves generation from periodic topography having various degree of roughness. Mercier *et al*. [8] applied the Hilbert transform to the physics of internal waves in two-dimensional fluids. Nakayama and Kakinuma [9] studied internal wave equations by employing simple iteration method. Benkhaldoun and Seaid [10] analyzed the shallow water equations by using simple finite volume method. Karunakar and Chakraverty [11] applied homotopy perturbation method to solve linear and nonlinear shallow water wave equation. Karunakar and Chakraverty [12] solved shallow water equation with crisp and uncertain initial conditions. Kounadis and Dougalis [13] implemented Galerkin finite element methods to solve shallow water equations over variable bottom. Jani and Singh [14] implemented Aboodh transform homotopy perturbation method to solve on fractional order model of atmospheric internal waves. Tamboli and Tandel [15] applied reduced differential transform method for the treatment of internal atmospheric waves phenomenon. Sahoo and Chakraverty [16] used Sawi transformn based homotopy perturbation method to solve shallow-water equation in fuzzy environment. Sartanpara and Meher [17] examined the differential equation system representing the atmospheric internal waves by using q-homotopy analysis Shehu transform method.

Fractional calculus has a strong history and plays a major role in the simulation of physical phenomenon of real life. Recently, there has been a lot of interest in the study of fractional calculus. Fractional calculus and fractional processes have become one of the most useful approaches to deal with a variety of problems in applied sciences due to memory and hereditary properties. A number of studies for fractional order linear, nonlinear, and complex dynamical mathematical models have been presented with interesting results during recent years. Therefore, compared to the traditional integer order models, fractional order models seem to be more objective and flexible.

The Caputo-Fabrizio (CF) fractional derivative appeared in 2015 [18] with the aim of avoiding singular kernal. Asjad *et al*. [19] studied heat transfer analysis of fractional second-grade fluid subject to Newtonian heating with CF fractional differential operator. Aydogan *et al*. [20] examined high order fractional integro-differential equations by employing CF fractional differential operator. Refai and Pal [21] examined linear and nonlinear fractional differential equations by applying CF fractional derivative of non-singular kernal. Shah and Khan [22] studied a fractional order heat transfer problem using Laplace transform by applying the CF fractional differential operator. Owolabi and Atangana [23] examined differential equations using a new three-step fractional Adams-Bashforth scheme by utilizing CF fractional differential operator. Shah *et al*. [24] studied fractional order telegraph equations using Elzaki Adomian decomposition method by utilizing Caputo derivative operator. Nisar *et al*. [25] examined bad impact of smoking in society by utilizing constant proportional-CF operator. Farman *et al*. [26] analyzed the cancer treatment with chemotherapy by using the fractal-fractional operator. Nisar *et al*. [27] studied a review on epidemic models in sight of fractional calculus. Farman *et al*. [28] studied system of fractional differential equations that represents a time-fractional order model. Farman *et al*. [29] worked on dynamic of infections in plant virus with fractal fractional operator. Farman *et al*. [30] studied measles epidemic model with the constant proportional Caputo operator. Some other useful results using fractional derivatives were presented in [31–33].

In this paper, a time-fractional order model of internal atmospheric waves with the CF fractional approach has been theoretically examined. The Elzaki Adomian decomposition method is used to solve the considered system of nonlinear partial differential equations. The unique analytical approximate solution for the given initial condition is determined. The numerical and graphical observations are presented for varying values of fractional parameter to depict the resulting variation in the solution. The considered model with CF time derivative is investigated for the time using the proposed technique in this work to the best of our knowledge and novel results have been obtained.

Elzaki Adomian decomposition method is a combination of the Elzaki transform and the Adomian decomposition method. Tariq Elzaki established the Elzaki transform in 2011 whereas the Adomian decomposition method was developed from 1970s to 1990s by George Adomian. Recently, Elzaki Adomian decomposition method has been successful to get the solution of different kinds of problems. Ziane and Cherif [34] applied Elzaki transform and Adomian decomposition method to solve nonlinear partial differential equations. Mamadu and Tsetimi [35] studied an approximation solution of integro-differential equation by applying Elzaki decomposition method. Ige *et al*. [36] worked on nonlinear fifth order Korteweg-de vries equations by applying Elzaki adomian decomposition method.

The rest of the paper has the following organization. The basic concepts and terms are recalled in Section 2. The considered model is described in Section 3. Section 4 contains the construction of solution of the problem by Alzaki Adomian decomposition. The numerical and graphical discussion is presented in Section 5. The results are discussed in Section 6. The last section presents the conclusion of the whole work.

## 2 Fundamental definitions and concepts

**Definition 1** [37] Let 0 < *α* < 1 and *v* be a continuously differentiable function. The CF fractional derivative of *v* of order *α* is given by

$$\begin{array}{c} D_{t}^{\alpha }v\left(t\right)=\frac{1}{1-\alpha }\int_{0}^{t}\;\text{exp}\;(-\frac{\alpha (t-s)}{1-\alpha })v^{\prime }\left(s\right)ds. \end{array}$$ (1)

**Definition 2** [38] The Elzaki transform is defined over the set of functions

$$\begin{array}{c} B=\{g\left(t\right)/\exists M,k_{1},k_{2}>0,|g\left(t\right)|<M\;\text{exp}\;(\frac{\left|t\right|}{k_{j}}),if\;t\in {\left(-1\right)}^{j}\times \left[0,\infty \right)\}, \end{array}$$ (2)

by the following integral

$$\begin{array}{c} E|g\left(t\right)|=T\left(s\right)=s\int_{0}^{\infty }g\left(t\right)\;\text{exp}\;(\frac{-t}{s})dt,t>0, \end{array}$$ (3)

where *s* is the factor of variable *t*.

Elzaki transform exhibits the following useful properties [38].

1. Convolution property

$$\begin{array}{c} E\left[f\left(t\right)*g\left(t\right)\right]=\frac{1}{s}E\left[f\left(t\right)\right]E\left[g\left(t\right)\right]. \end{array}$$ (4)

2. Differentiation property

If *f*^(*m*)^(*t*) is the *m* − *th* derivative of the function *f*(*t*) ∈ *B* with respect to *t* then its Elzaki transform is given by

$$\begin{array}{c} \text{E}[f^{\left(m\right)}\left(t\right)]=\frac{1}{s^{m}}T\left(s\right)-\sum_{n=0}^{m-1}s^{2-m+n}f^{\left(n\right)}\left(0\right). \end{array}$$ (5)

**Theorem 3** [39] The Elzaki transform of the CF fractional derivative is represented, as

$$\begin{array}{c} E[D_{t}^{\alpha }\left(f\left(t\right)\right)]=\frac{s(\frac{F(s)}{s}-sf\left(0\right))}{1-\alpha (1-s)}. \end{array}$$ (6)

In general,

$$\begin{array}{c} E[D_{t}^{m+\alpha }\left(f\left(t\right)\right)]=\frac{s(\frac{F(s)}{s^{m+1}}-\sum_{n=0}^{m}s^{1-m+n}f^{\left(n\right)}\left(0\right))}{1-\alpha (1-s)}. \end{array}$$ (7)

## 3 Governing model

A set of nonlinear partial differential equations based on the shallow fluid hypothesis are used to describe atmospheric internal waves. Utilising the conservation of mass and momentum, the primary equations of fluid motion are determined in differential form. As the name implies, the wave-length of shallow fluid is greater than the depth of the fluid layer. The central momentum equations are as follows [40]:

$$\begin{array}{c} \frac{\partial \lambda }{\partial t}+\lambda \frac{\partial \lambda }{\partial x}+\phi \frac{\partial \lambda }{\partial y}+n\frac{\partial \lambda }{\partial z}-w\phi +\frac{1}{\rho }\frac{\partial \tilde{\phi }}{\partial x}=0, \end{array}$$ (8)

$$\begin{array}{c} \frac{\partial \phi }{\partial t}+\lambda \frac{\partial \phi }{\partial x}+\phi \frac{\partial \phi }{\partial y}+n\frac{\partial \phi }{\partial z}+w\lambda +\frac{1}{\rho }\frac{\partial \tilde{\phi }}{\partial y}=0, \end{array}$$ (9)

$$\begin{array}{c} \frac{\partial \tilde{\phi }}{\partial z}=-\rho r. \end{array}$$ (10)

Assuming that the atmosphere is a homogeneous and incompressible fluid, the equation of continuity can be written, as

$$\begin{array}{c} \frac{\partial \lambda }{\partial x}+\frac{\partial \phi }{\partial y}+\frac{\partial n}{\partial z}=0, \end{array}$$ (11)

and the equation of hydrostatic can be expressed in the form

$$\begin{array}{c} \frac{\partial \tilde{\phi }}{\partial z}=-\rho_{0}r, \end{array}$$ (12)

where *ρ*_0_ is constant.

Differentiating Eq (12) with respect to *x*, the following equation is derived.

$$\begin{array}{c} \frac{\partial }{\partial x}(\frac{\partial \tilde{\phi }}{\partial z})=\frac{\partial }{\partial z}(\frac{\partial \tilde{\phi }}{\partial x})=0. \end{array}$$ (13)

If *U*_*T*_ and *U*_*B*_ are the pressure at the fluid’s top and bottom boundaries, respectively, then the integration of Eq (12) through the depth of the fluid yields

$$\begin{array}{c} \int_{z(U_{B})}^{z(U_{T})}\frac{\partial \tilde{\phi }}{\partial z}dz=-\rho_{0}r\int_{z(U_{B})}^{z(U_{T})}dz, \end{array}$$ (14)

or

$$\begin{array}{c} U_{B}-U_{T}=r\rho_{0}\psi , \end{array}$$ (15)

*ψ* being the depth of the fluid. If *U*_*T*_ = 0 or *U*_*T*_ << *U*_*B*_,

$$\begin{array}{c} \frac{U_{B}}{\rho_{0}}=r\psi , \end{array}$$ (16)

and

$$\begin{array}{c} \frac{1}{\rho_{0}}\frac{\partial U_{B}}{\partial x}=r\frac{\partial \psi }{\partial x}. \end{array}$$ (17)

According to the supposition that the fluid’s bottom gradient is proportionate to the horizontal pressure gradient at its base, a new form of pressure-gradient appearing in Eqs (8) and (9) can be gained. Integrating the incompressible continuity equation Eq (11) with respect to *z*, it can be written, as

$$\begin{array}{c} \int_{0}^{z}\frac{\partial n}{\partial z}dz=-\int_{0}^{z}(\frac{\partial \lambda }{\partial x}+\frac{\partial \phi }{\partial y})dz. \end{array}$$ (18)

Given that the pressure gradient is not a function of *z*, the derivatives of λ and *ϕ* would also not be functions of z if they are not initially. This gives

$$\begin{array}{c} n\left(z\right)-n\left(0\right)=-(\frac{\partial \lambda }{\partial x}+\frac{\partial \phi }{\partial y})\psi , \end{array}$$ (19)

for *z* = *ψ*. The base of the fluid has 0 vertical velocity. Also,

$$\begin{array}{c} n\left(z\right)=\frac{Dz}{Dt}{|}_{\psi }=\frac{D\psi }{Dt}. \end{array}$$ (20)

Hence, the equation for the shallow-water continuity becomes

$$\begin{array}{c} \frac{D\psi }{Dt}=-\psi (\frac{\partial \lambda }{\partial x}+\frac{\partial Q}{\partial y}). \end{array}$$ (21)

In terms of λ, *ϕ* and *ψ*, the following system of three equations is obtained.

$$\begin{array}{c} \begin{array}{ccc} \frac{\partial \lambda }{\partial t}+\lambda \frac{\partial \lambda }{\partial x}+\phi \frac{\partial \lambda }{\partial y}-w\phi +r\frac{\partial \psi }{\partial x} & = & 0,\\ \frac{\partial \phi }{\partial t}+\lambda \frac{\partial \phi }{\partial x}+\phi \frac{\partial \phi }{\partial y}+w\lambda +r\frac{\partial \psi }{\partial y} & = & 0,\\ \frac{\partial \psi }{\partial t}+\lambda \frac{\partial \psi }{\partial x}+\phi \frac{\partial \psi }{\partial y}+\psi (\frac{\partial \lambda }{\partial x}+\frac{\partial \phi }{\partial y}) & = & 0. \end{array} \end{array}$$ (22)

If the one-dimensional equations in terms of λ, *ϕ* and *ψ* are considered and a mean component is provided on which perturbations arise by clarifying a continuous pressure-gradient of desired magnitude in the *y* direction, then the system of nonlinear partial differential equations becomes

$$\begin{array}{c} \begin{array}{ccc} \frac{\partial \lambda }{\partial t}+\lambda \frac{\partial \lambda }{\partial x}-w\phi +r\frac{\partial \psi }{\partial x} & = & 0,\\ \frac{\partial \phi }{\partial t}+\lambda \frac{\partial \phi }{\partial x}+w\lambda +r\bar{L} & = & 0,\\ \frac{\partial \psi }{\partial t}+\lambda \frac{\partial \psi }{\partial x}+\phi \bar{L}+\psi \frac{\partial \lambda }{\partial x} & = & 0, \end{array} \end{array}$$ (23)

where

$$\begin{array}{ccc} \bar{L} & = & -\frac{w}{r}\bar{V}. \end{array}$$ (24)

The time-fractional order model can be written, as

$$\begin{array}{c} \begin{array}{ccc} D_{t}^{\alpha }\lambda +\lambda \frac{\partial \lambda }{\partial x}-w\phi +r\frac{\partial \psi }{\partial x} & = & 0,\\ D_{t}^{\alpha }\phi +\lambda \frac{\partial \phi }{\partial x}+w\lambda +r\bar{L} & = & 0,\\ D_{t}^{\alpha }\psi +\lambda \frac{\partial \psi }{\partial x}+\phi \bar{L}+\psi \frac{\partial \lambda }{\partial x} & = & 0, \end{array} \end{array}$$ (25)

where $D_{t}^{\alpha }$ is the time-fractional differential operator as defined in Section 3, *x* is spacial coordinate, *t* is temporal coordinate, the independent variables λ and *ϕ* are cartesian velocities, *ψ* means fluid depth, *w* means the Coriolis parameter, *r* is gravity acceleration, $\bar{L}$ shows mean fluid depth and $\bar{V}$ shows the specified, constant mean geostrophic speed [2, 40].

Here, the following fix parameters in calculations are employed. Coriolis parameter *w* = 2Ω sin*α*, where Ω = 7.29 × 10^−5^rad/s rad/s and $\alpha =\frac{\pi }{3}$, constant of gravity *r* = 9.8*m*/*s*^2^ and $\bar{V}=2.5m/s$.

## 4 Mathematical analysis

In this section the Elzaki transform is applied in combination with the Adomian decomposition considering the fractional derivative by Caputo-Fabrizio approach.

To determine the unique solution for the time-fractional model expressed by (25), the initial conditions are considered, as [2]

$$\begin{array}{c} \begin{array}{ccc} \lambda (x,0) & = & e^{x}{\text{sech}}^{2}\left(x\right),\\ \phi (x,0) & = & 2x{\text{sech}}^{2}\left(2x\right),\\ \psi (x,0) & = & x^{2}{\text{sech}}^{2}\left(2x\right). \end{array} \end{array}$$ (26)

Applying the Elzaki transform on Eq (25), the system can be expressed, as

$$\begin{array}{c} \begin{array}{ccc} \text{E}[D_{t}^{\alpha }\lambda \left(x,t\right)] & = & -\text{E}[\lambda \frac{\partial \lambda }{\partial x}]+\text{E}\left[w\phi \right]-\text{E}[r\frac{\partial \psi }{\partial x}],\\ \text{E}[D_{t}^{\alpha }\phi \left(x,t\right)] & = & -\text{E}[\lambda \frac{\partial \phi }{\partial x}]-\text{E}\left[w\lambda \right]-\text{E}\left[r\bar{L}\right],\\ \text{E}[D_{t}^{\alpha }\psi \left(x,t\right)] & = & -\text{E}[\lambda \frac{\partial \psi }{\partial x}]-\text{E}\left[\phi \bar{L}\right]-\text{E}[\psi \frac{\partial \lambda }{\partial x}]. \end{array} \end{array}$$ (27)

Taking into account the CF fractional derivative, (27) yields

$$\begin{array}{c} \begin{array}{ccc} \frac{\lambda \left(x,s\right)-s^{2}\lambda \left(x,0\right)}{1-\alpha (1-s)} & = & -\text{E}[\lambda \frac{\partial \lambda }{\partial x}]+\text{E}\left[w\phi \right]-\text{E}[r\frac{\partial \psi }{\partial x}],\\ \frac{\phi \left(x,s\right)-s^{2}\phi \left(x,0\right)}{1-\alpha (1-s)} & = & -\text{E}[\lambda \frac{\partial \phi }{\partial x}]-\text{E}\left[w\lambda \right]-\left[r\bar{L}s^{2}\right],\\ \frac{\psi \left(x,s\right)-s^{2}\psi \left(x,0\right)}{1-\alpha (1-s)} & = & -\text{E}[\lambda \frac{\partial \psi }{\partial x}]-\text{E}\left[\phi \bar{L}\right]-\text{E}[\psi \frac{\partial \lambda }{\partial x}]. \end{array} \end{array}$$ (28)

By using the initial conditions and then applying the inverse Elzaki transform, the following set of equations is retrieved.

$$\begin{array}{ccc} \lambda (x,t) & = & e^{x}{\text{sech}}^{2}\left(x\right)-{\text{E}}^{-1}[1-\alpha \left(1-s\right)\text{E}(\lambda \frac{\partial \lambda }{\partial x})]+{\text{E}}^{-1}\left[1-\alpha \left(1-s\right)w\text{E}\left(\phi \left(x,t\right)\right)\right]\\ & & -{\text{E}}^{-1}[1-\alpha \left(1-s\right)\text{E}(r\frac{\partial \psi }{\partial x})], \end{array}$$ (29)

$$\begin{array}{ccc} \phi (x,t) & = & 2x{\text{sech}}^{2}\left(2x\right)-{\text{E}}^{-1}[1-\alpha \left(1-s\right)\text{E}(\lambda \frac{\partial \phi }{\partial x})]-{\text{E}}^{-1}[1-\alpha (1-s)w\text{E}(\lambda (x,t))]\\ & & -[r\bar{L}\left(1-\alpha \left(1-t\right)\right)], \end{array}$$ (30)

$$\begin{array}{ccc} \psi (x,t) & = & x^{2}{\text{sech}}^{2}\left(2x\right)-{\text{E}}^{-1}[1-\alpha \left(1-s\right)\text{E}(\lambda \frac{\partial \psi }{\partial x})]-{\text{E}}^{-1}\left[1-\alpha \left(1-s\right)\text{E}\left(\phi \bar{L}\right)\right]\\ & & -{\text{E}}^{-1}[1-\alpha \left(1-s\right)\text{E}(\psi \frac{\partial \lambda }{\partial x})]. \end{array}$$ (31)

Applying Adomian decomposition, the afore-mentioned set can be expressed, as

$$\begin{array}{ccc} \sum_{n=0}^{\infty }\lambda_{n}\left(x,t\right) & = & e^{x}{\text{sech}}^{2}\left(x\right)-{\text{E}}^{-1}[1-\alpha \left(1-s\right)\text{E}(\sum_{n=0}^{\infty }X_{n})]\\ & & +{\text{E}}^{-1}[1-\alpha \left(1-s\right)w\text{E}(\sum_{n=0}^{\infty }\phi_{n})]-{\text{E}}^{-1}[1-\alpha \left(1-s\right)r\text{E}(\sum_{n=0}^{\infty }Z_{n})], \end{array}$$ (32)

$$\begin{array}{ccc} \sum_{n=0}^{\infty }\phi_{n}\left(x,t\right) & = & 2x{\text{sech}}^{2}\left(2x\right)-{\text{E}}^{-1}[1-\alpha \left(1-s\right)\text{E}(\sum_{n=0}^{\infty }Y_{n})]\\ & & -{\text{E}}^{-1}[1-\alpha \left(1-s\right)w\text{E}(\sum_{n=0}^{\infty }\lambda_{n})]-r\bar{L}\left(1-\alpha +\alpha t\right), \end{array}$$ (33)

$$\begin{array}{ccc} \sum_{n=0}^{\infty }\psi_{n}\left(x,t\right) & = & x^{2}{\text{sech}}^{2}\left(2x\right)-{\text{E}}^{-1}[1-\alpha \left(1-s\right)\text{E}(\sum_{n=0}^{\infty }A_{n})]\\ & & -{\text{E}}^{-1}[1-\alpha \left(1-s\right)\bar{L}\text{E}(\sum_{n=0}^{\infty }\phi_{n})]-{\text{E}}^{-1}[1-\alpha \left(1-s\right)\text{E}(\sum_{n=0}^{\infty }B_{n})]. \end{array}$$ (34)

Comparing the results on both sides of Eq (32), the following recurrence relation is determined.

$$\begin{array}{ccc} \lambda_{0} & = & e^{x}{\text{sech}}^{2}\left(x\right), \end{array}$$ (35)

$$\begin{array}{ccc} \lambda_{n+1} & = & -{\text{E}}^{-1}\left[1-\alpha \left(1-s\right)\text{E}\left(X_{n}\right)\right]+{\text{E}}^{-1}\left[1-\alpha \left(1-s\right)w\text{E}\left(\phi_{n}\right)\right]\\ & & -{\text{E}}^{-1}\left[1-\alpha \left(1-s\right)r\text{E}\left(Z_{n}\right)\right],\;\;\;n=0,\;1,\;2,.... \end{array}$$ (36)

Comparison of both sides of Eq (33) implies

$$\begin{array}{ccc} \phi_{0} & = & 2x{\text{sech}}^{2}\left(2x\right), \end{array}$$ (37)

$$\begin{array}{ccc} \phi_{n+1} & = & -{\text{E}}^{-1}\left[1-\alpha \left(1-s\right)\text{E}\left(Y_{n}\right)\right]-{\text{E}}^{-1}\left[1-\alpha \left(1-s\right)w\text{E}\left(\lambda_{n}\right)\right]\\ & & -r\bar{L}\left(1-\alpha +\alpha t\right),\;\;\;n=1,\;2,\;3.... \end{array}$$ (38)

Similarly, Eq (34) yields

$$\begin{array}{ccc} \psi_{0} & = & x^{2}{\text{sech}}^{2}\left(2x\right), \end{array}$$ (39)

$$\begin{array}{ccc} \psi_{n+1} & = & -{\text{E}}^{-1}\left[1-\alpha \left(1-s\right)\text{E}\left(A_{n}\right)\right]-{\text{E}}^{-1}\left[1-\alpha \left(1-s\right)\bar{L}\text{E}\left(\phi_{n}\right)\right]\\ & & -{\text{E}}^{-1}\left[1-\alpha \left(1-s\right)\text{E}\left(B_{n}\right)\right],\;\;\;n=1,\;2,\;3,..., \end{array}$$ (40)

where *X*_*n*_, *Z*_*n*_, *Y*_*n*_, *A*_*n*_ and *B*_*n*_ are the Adomian polynomials given by λλ_*x*_, *ψ*_*x*_, λ*ϕ*_*x*_, λ*ψ*_*x*_ and *ψ*λ_*x*_ respectively.

The successive Adomian polynomials are calculated, as follows:

$$\begin{array}{ccc} X_{0} & = & \lambda_{0}\lambda_{0x},\;X_{1}=\lambda_{1}\lambda_{0x}+\lambda_{0}\lambda_{1x},\;X_{2}=\lambda_{2}\lambda_{0x}+\lambda_{1}\lambda_{1x}+\lambda_{0}\lambda_{2x},..., \end{array}$$ (41)

$$\begin{array}{ccc} Z_{0} & = & \psi_{0x},\;Z_{1}=\psi_{1x},..., \end{array}$$ (42)

$$\begin{array}{ccc} Y_{0} & = & \lambda_{0}\phi_{0x},\;Y_{1}=\lambda_{0}\phi_{1x}+\lambda_{1}\phi_{0x},..., \end{array}$$ (43)

$$\begin{array}{ccc} A_{0} & = & \lambda_{0}\psi_{0x},\;A_{1}=\lambda_{0}\psi_{1x}+\lambda_{1}\psi_{0x},..., \end{array}$$ (44)

$$\begin{array}{ccc} B_{0} & = & \psi_{0}\lambda_{0x},\;B_{1}=\psi_{0}\psi_{1x}+\psi_{1}\lambda_{0x},...,\\ & \vdots & \end{array}$$ (45)

Using the recurrence relations given by Eqs (35)–(40) along with the Adomian polynomials defined by Eqs (41)–(45), the following results are determined.

$$\begin{array}{ccc} \lambda_{0} & = & e^{x}{\text{sech}}^{2}\left(x\right), \end{array}$$ (46)

$$\begin{array}{ccc} \lambda_{1} & = & \left(1-\alpha +\alpha t\right)(-e^{2x}{\text{sech}}^{4}\left(x\right)+2e^{2x}{\text{sech}}^{4}\left(x\right)\;\text{tanh}\left(x\right)+2xw{\text{sech}}^{2}\left(2x\right)\\ & & -2xr{\text{sech}}^{2}\left(2x\right)+4x^{2}r{\text{sech}}^{2}\left(2x\right)\;\text{tanh}\left(2x\right)), \end{array}$$ (47)

$$\begin{array}{ccc} \phi_{0} & = & 2x{\text{sech}}^{2}\left(2x\right), \end{array}$$ (48)

$$\begin{array}{ccc} \phi_{1} & = & \left(1-\alpha +\alpha t\right)(-2e^{x}{\text{sech}}^{2}\left(x\right){\text{sech}}^{2}\left(2x\right)+8xe^{x}{\text{sech}}^{2}\left(x\right){\text{sech}}^{2}\left(2x\right)\;\text{tanh}\left(2x\right)\\ & & -we^{x}{\text{sech}}^{2}\left(x\right)-r\bar{L}), \end{array}$$ (49)

$$\begin{array}{ccc} \psi_{0} & = & x^{2}{\text{sech}}^{2}\left(2x\right), \end{array}$$ (50)

$$\begin{array}{ccc} \psi_{1} & = & \left(1-\alpha +\alpha t\right)(-2xe^{x}{\text{sech}}^{2}\left(x\right){\text{sech}}^{2}\left(2x\right)+4x^{2}e^{x}{\text{sech}}^{2}\left(x\right){\text{sech}}^{2}\left(2x\right)\;\text{tanh}\left(2x\right)\\ & & -x^{2}e^{x}{\text{sech}}^{2}\left(x\right){\text{sech}}^{2}\left(2x\right)+2e^{x}x^{2}{\text{sech}}^{2}\left(x\right){\text{sech}}^{2}\left(2x\right)\;\text{tanh}\left(x\right)\\ & & +0.0000644217x{\text{sech}}^{2}\left(2x\right)),\\ & \vdots & \end{array}$$ (51)

Hence, the functions λ(*x*, *t*), *ϕ*(*x*, *t*) and *ψ*(*x*, *t*) are determined, as

$$\begin{array}{ccc} \lambda (x,t) & = & e^{x}{\text{sech}}^{2}\left(x\right)+\left(1-\alpha +\alpha t\right)(-19.5997x{\text{sech}}^{2}\left(2x\right)+e^{2x}{\text{sech}}^{4}\left(x\right)\left(-1+2\;\text{tanh}\left(x\right)\right)\\ & & +39.2x^{2}{\text{sech}}^{2}\left(2x\right)\;\text{tanh}\left(2x\right))+\cdots , \end{array}$$ (52)

$$\begin{array}{ccc} \phi (x,t) & = & 2x{\text{sech}}^{2}\left(2x\right)+\left(1-\alpha +\alpha t\right)(0.0003156662597+e^{x}{\text{sech}}^{2}\left(x\right)\\ & & (-0.000126267+{\text{sech}}^{2}\left(2x\right)\left(-2+8x\;\text{tanh}\left(2x\right)\right))+\cdots , \end{array}$$ (53)

$$\begin{array}{ccc} \psi (x,t) & = & x^{2}{\text{sech}}^{2}\left(2x\right)+\left(1-\alpha +\alpha t\right)(-2xe^{x}{\text{sech}}^{2}\left(x\right){\text{sech}}^{2}\left(2x\right)\\ & & +4x^{2}e^{x}{\text{sech}}^{2}\left(x\right){\text{sech}}^{2}\left(2x\right)\;\text{tanh}\left(2x\right)-x^{2}e^{x}{\text{sech}}^{2}\left(x\right){\text{sech}}^{2}\\ & & +2e^{x}x^{2}{\text{sech}}^{2}\left(x\right){\text{sech}}^{2}\left(2x\right)\;\text{tanh}\left(x\right)+0.0000644217x{\text{sech}}^{2}\left(2x\right))+\cdots , \end{array}$$ (54)

## 5 Numerical and graphical observations

In this section, the numerical and graphical results for the system of equations describing the internal waves in the atmosphere with CF approach are presented. The influence of the CF fractional parameter *α* is presented by observing the solution for distinct values of *α*. The calculations and simulations are performed using MATHEMATICA software.

Tables 1 and 2 show the numerical results for the solution at time 0.1 for *α* = 0.6 and *α* = 0.8, respectively. The numerical values of the solution at time 0.04 are summarized as shown in Tables 3–7 for different values of *α*. The numerical solutions at *t* = 0.04 with *α* = 1 are shown along side the results obtained through the homotopy analysis and Elazaki Adomian decomposition techniques [2, 41]. It is evident that the results presented in this work are in good agreement with those available in literature.

**Table 1:** Approximate solutions at *t* = 0.1 and *α* = 0.6.

*x*	λ	*ϕ*	*ψ*
0	0.54	−0.919913	0
0.2	−0.518171	−0.300821	−0.133672
0.4	0.1514112	0.488321	−0.0462589
0.6	1.35454	0.730218	0.11475
0.8	1.9932	0.576793	0.179859
1.0	2.04643	0.353313	0.158929
1.2	1.8043	0.190095	0.1104
1.4	1.4787	0.0951829	0.0672173
1.6	1.17042	0.0457387	0.0377796
1.8	0.913391	0.021481	0.0201763
2	0.710862	0.00998345	0.01042

**Table 2:** Approximate solutions at *t* = 0.1 and *α* = 0.8.

*x*	λ	*ϕ*	*ψ*
0	0.72	−0.559947	0
0.2	0.143913	−0.0491824	−0.0679728
0.4	0.591649	0.472247	0.00684414
0.6	1.33186	0.587708	0.112816
0.8	1.70011	0.445335	0.147177
1.0	1.69237	0.270352	0.124385
1.2	1.49455	0.146123	0.0854473
1.4	1.24308	0.0740245	0.0521758
1.6	1.00417	0.0361356	0.029632
1.8	0.801126	0.0172759	0.0160618
2	0.636977	0.00817576	0.00844149

**Table 3:** Approximate solutions at *t* = 0.04 and *α* = 0.6.

*x*	λ	*ϕ*	*ψ*
0	0.576	−0.84792	0
0.2	−0.385754	−0.250493	−0.120532
0.4	0.23946	0.485106	−0.0356383
0.6	1.35001	0.701716	0.114363
0.8	1.93458	0.550501	0.173323
1.0	1.97562	0.336721	0.15202
1.2	1.74235	0.1813	0.105409
1.4	1.43158	0.0909512	0.064209
1.6	1.13717	0.0438181	0.0361501
1.8	0.890938	0.02064	0.0193534
2	0.696085	0.00962191	0.0100243

**Table 4:** Approximate solutions at *t* = 0.04 and *α* = 0.8.

*x*	λ	*ϕ*	*ψ*
0	0.768	−0.463956	0
0.2	0.320468	0.0179212	−0.050453
0.4	0.709046	0.467961	0.0210049
0.6	1.32581	0.549705	0.1123
0.8	1.62195	0.410279	0.138462
1.0	1.59795	0.248229	0.115174
1.2	1.41194	0.134397	0.0787934
1.4	1.18024	0.0683823	0.0481648
1.6	0.95984	0.0335748	0.0274593
1.8	0.771189	0.0161546	0.0149645
2	0.617274	0.00769371	0.00791389

**Table 5:** Numerical comparison of solution of λ at *t* = 0.04 and *α* = 1.

*x*	Proposed method	*EADM*	*HAM*
0	0.96	0.96	0.967
0.2	1.02669082	1.02669	1.05244
0.4	1.17863747	1.17863	1.19575
0.6	1.30162028	1.30162	1.30074
0.8	1.30933028	1.30933	1.29793
1.0	1.22029028	1.22029	1.20652
1.2	1.08153486	1.08154	1.06949
1.4	0.92890202	0.928903	0.91974
1.6	0.78251393	0.782509	0.776044
1.8	0.65143778	0.651439	0.647074
2	0.53845909	0.538462	0.535589

**Table 6:** Numerical comparison of solution of *ϕ* at *t* = 0.04 and *α* = 1.

*x*	Proposed method	*EADM*	*HAM*
0	−0.07999242404	−0.0799924	−0.0659938
0.2	0.2863362968	0.286336	0.296122
0.4	0.4508158959	0.450816	0.450193
0.6	0.3976930803	0.397693	0.392154
0.8	0.2700560311	0.270056	0.264948
1.0	0.1597377842	0.159737	0.156515
1.2	0.08749271913	0.0874927	0.0857864
1.4	0.04581335932	0.0458133	0.0449939
1.6	0.02333142066	0.0233315	0.022961
1.8	0.01166924925	0.0116692	0.0115083
2	0.005765508494	0.00576551	0.00569735

**Table 7:** Numerical comparison of solution of *ψ* at *t* = 0.04 and *α* = 1.

*x*	Proposed method	*EADM*	*HAM*
0	0	0	0
0.2	0.01962584234	0.0196258	0.0221808
0.4	0.07764813085	0.0776482	0.0797133
0.6	0.110239781	0.110237	0.110162
0.8	0.1036027938	0.1036	0.102329
1.0	0.078326331	0.0783272	0.0769838
1.2	0.05217495704	0.0521776	0.0512072
1.4	0.03212254212	0.0321206	0.0315356
1.6	0.01877056	0.0187685	0.0184517
1.8	0.01057434816	0.0105757	0.0104157
2	0.00579966984	0.00580347	0.00572653

**Fig 1:**
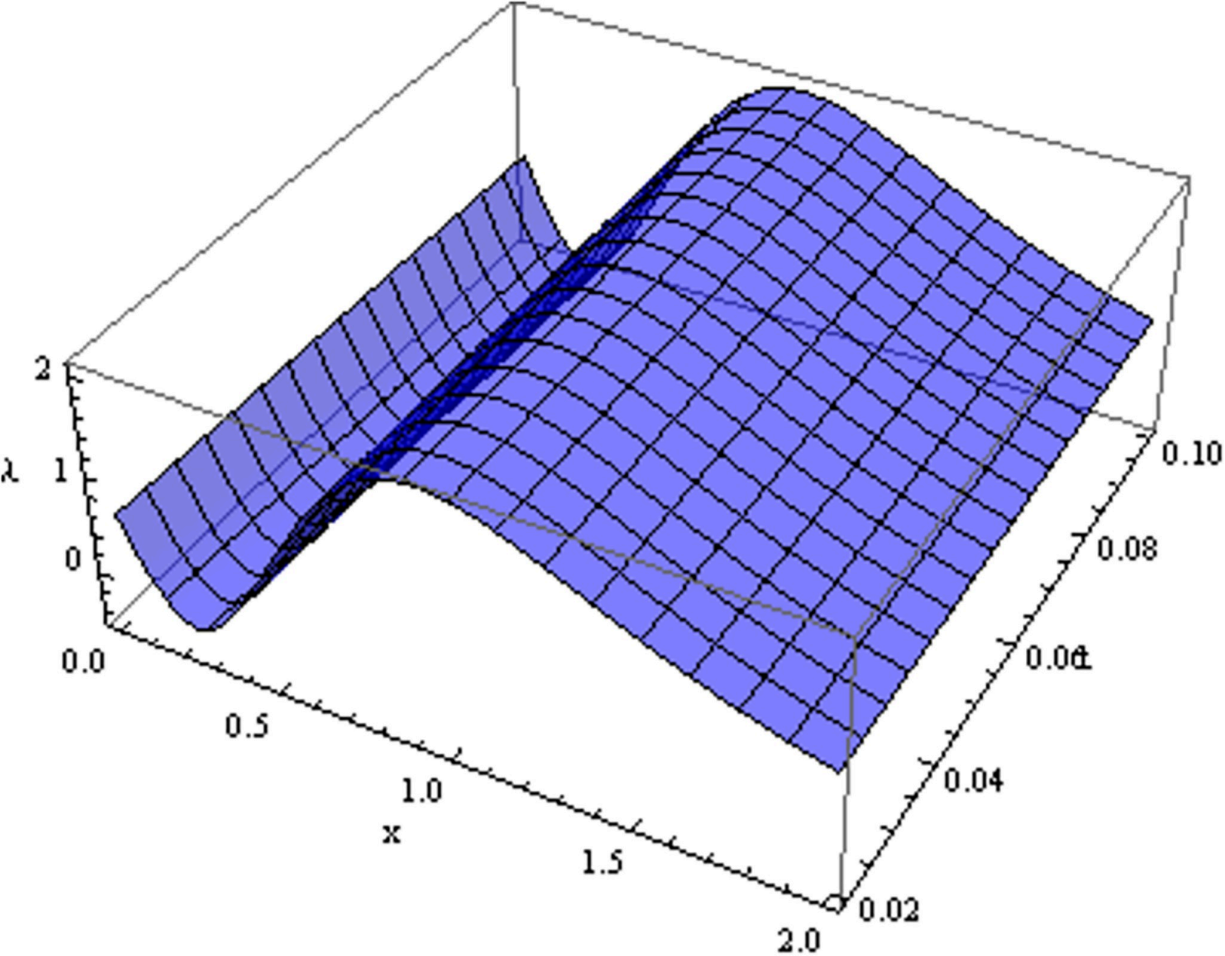
Variation of λ with change in *α*: *α* = 0.6.

**Fig 2:**
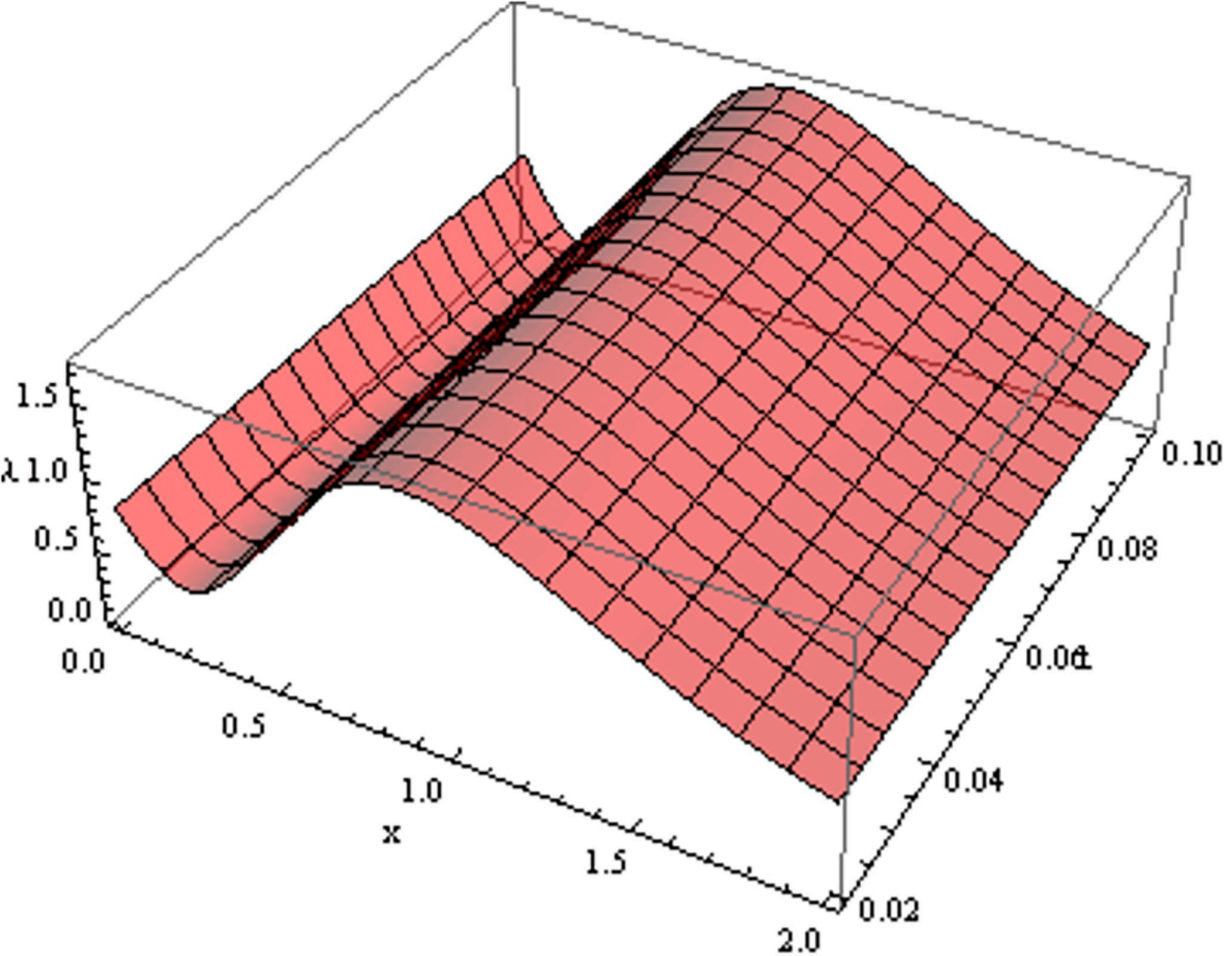
Variation of λ with change in *α*: *α* = 0.8.

**Fig 3:**
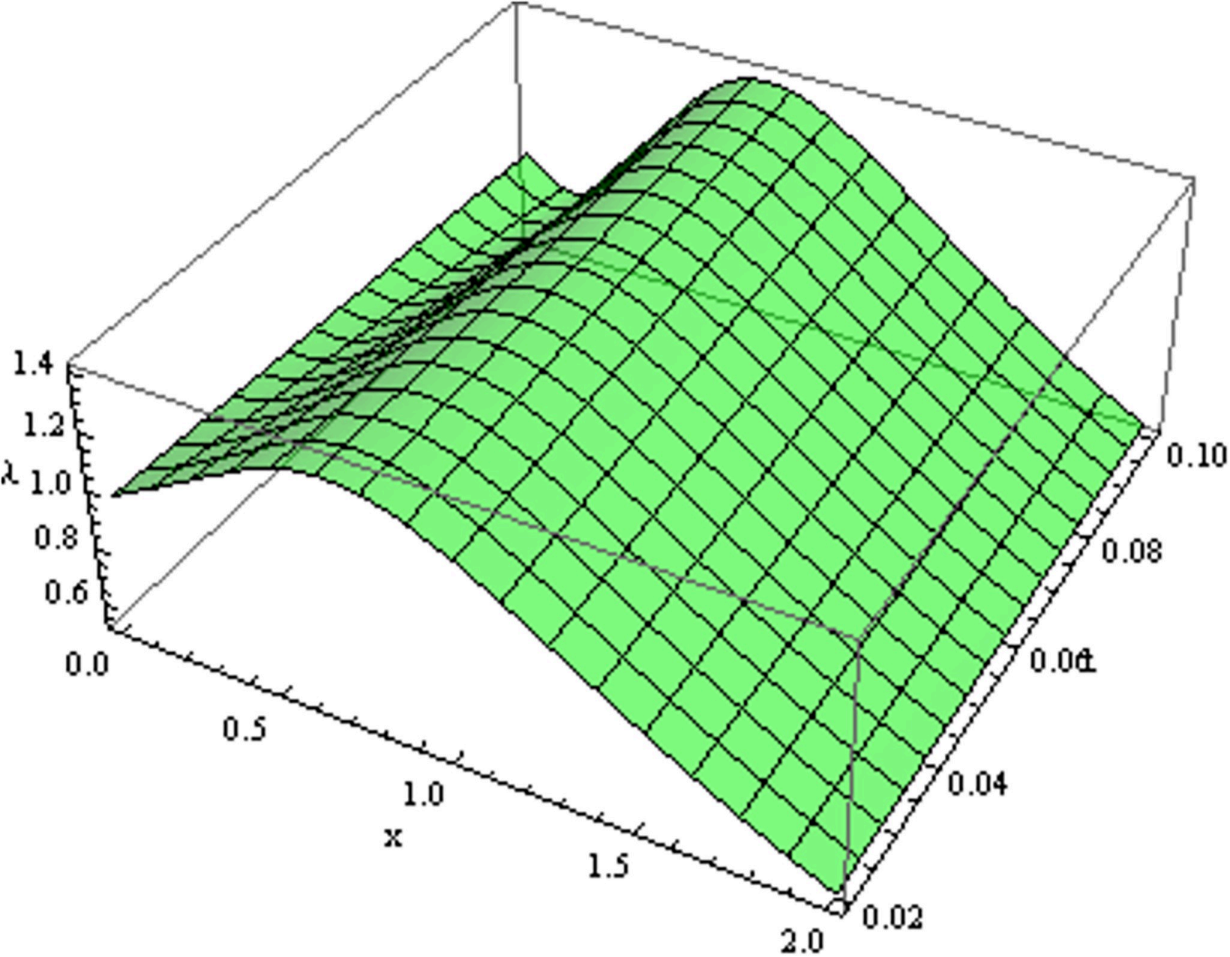
Variation of λ with change in *α*: *α* = 1.

**Fig 4:**
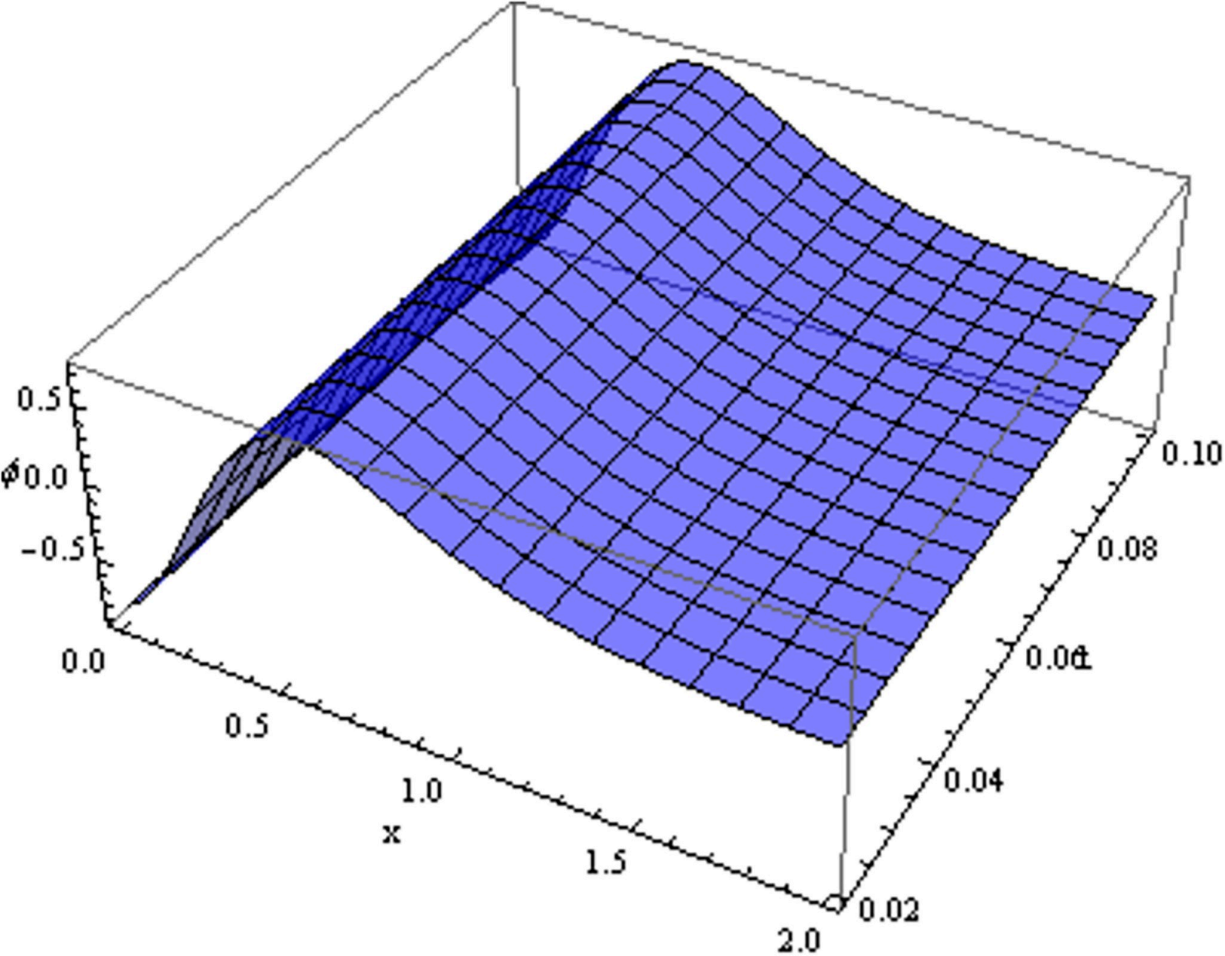
Variation of *ϕ* with change in *α*: *α* = 0.6.

**Fig 5:**
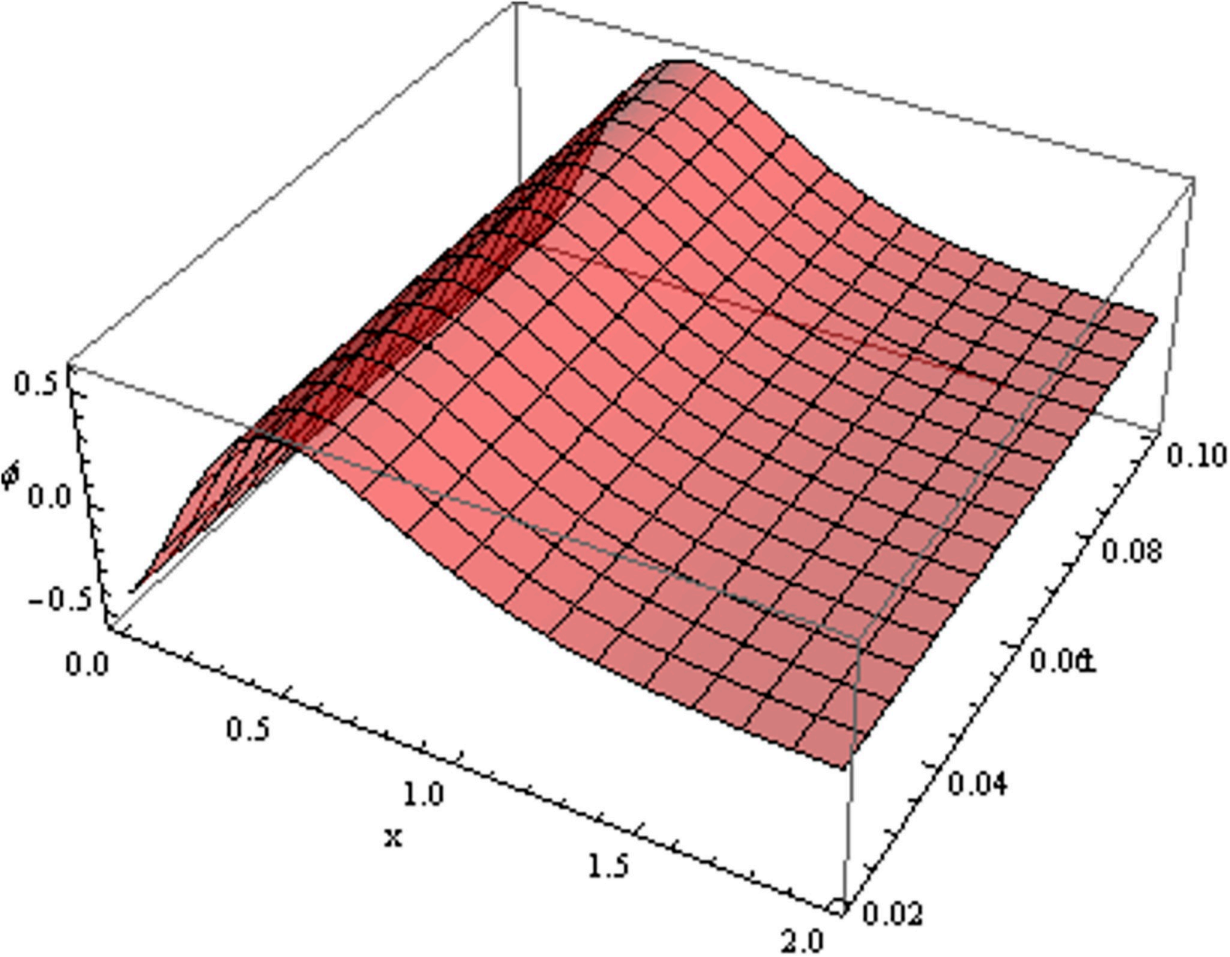
Variation of *ϕ* with change in *α*: *α* = 0.8.

**Fig 6:**
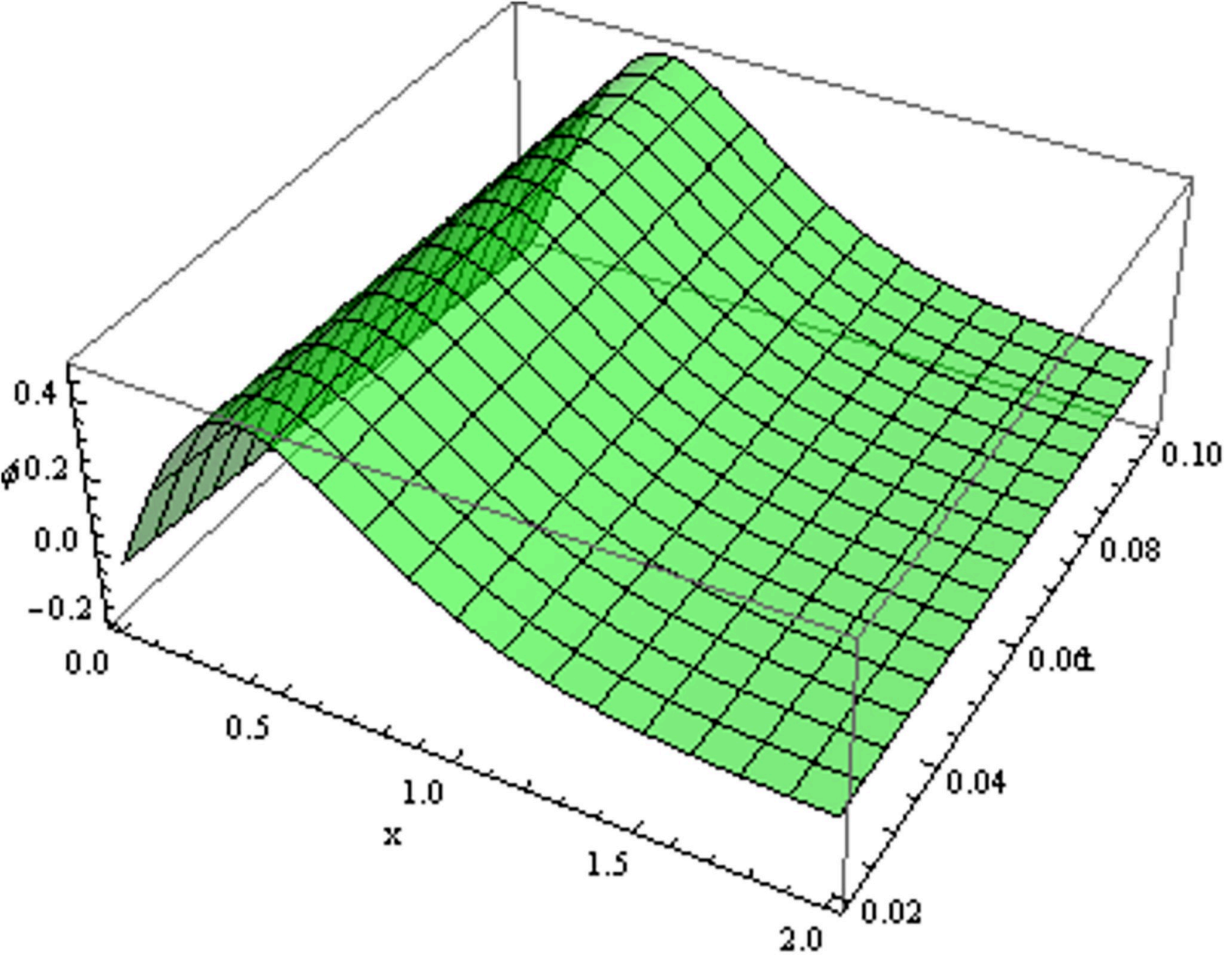
Variation of *ϕ* with change in *α*: *α* = 1.

**Fig 7:**
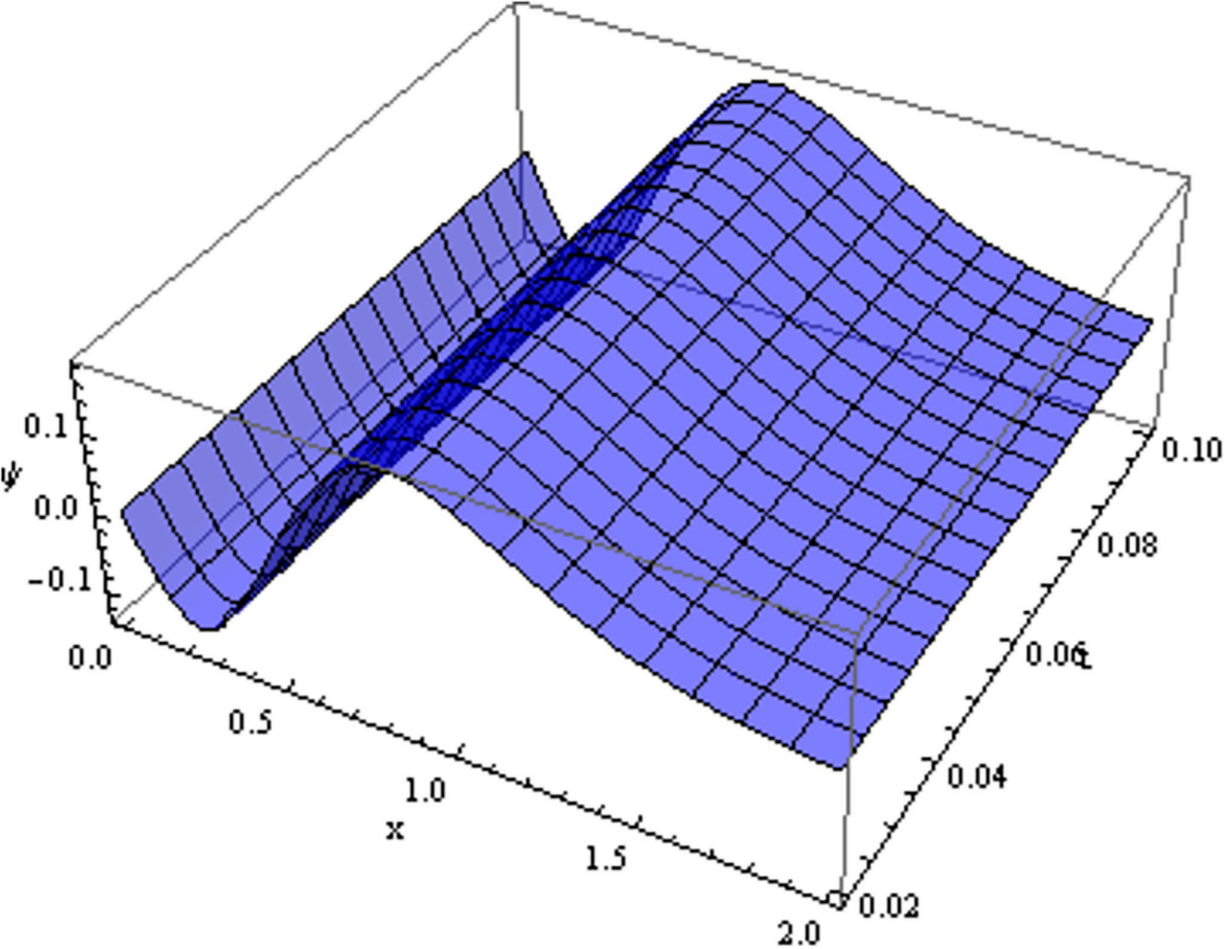
Variation of *ψ* with change in *α*: *α* = 0.6.

**Fig 8:**
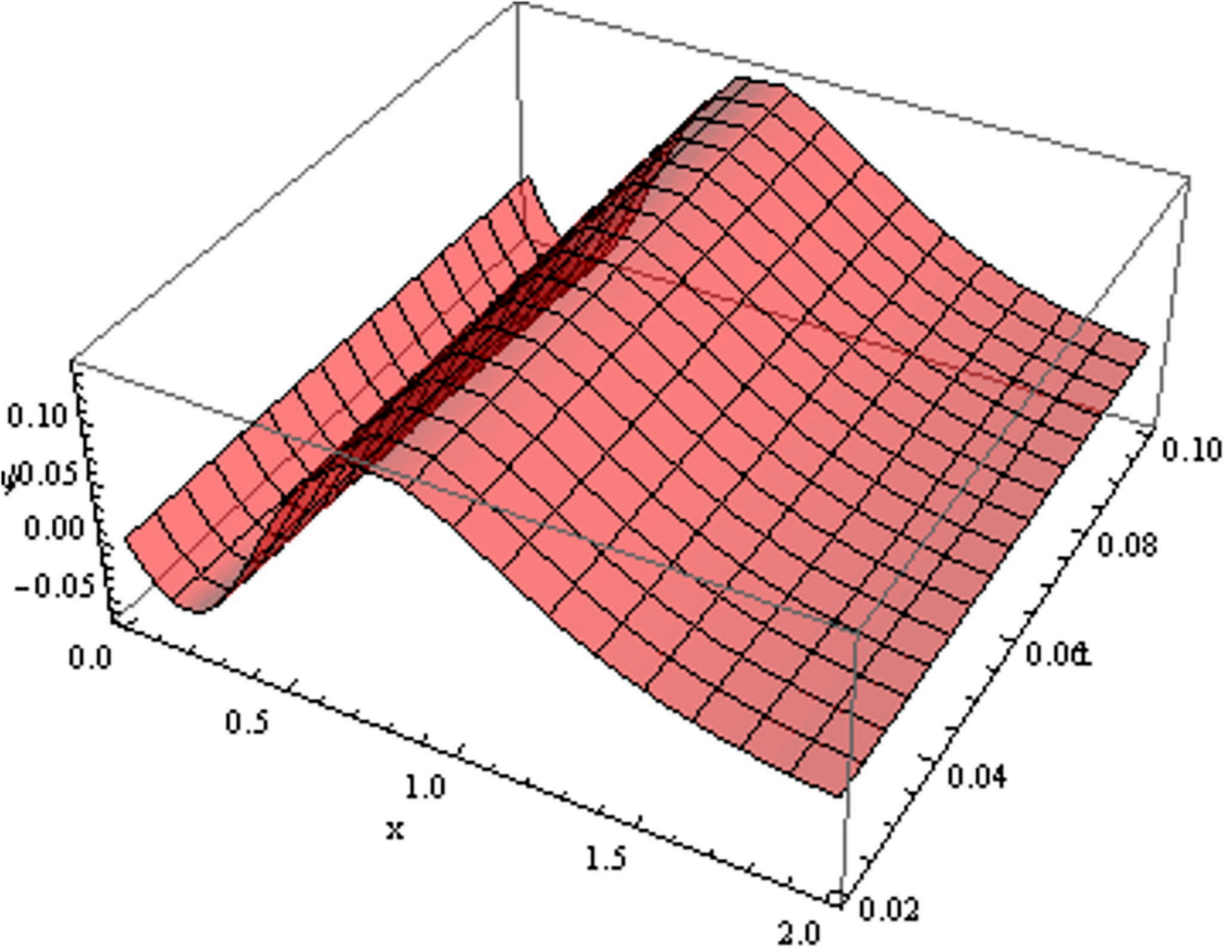
Variation of *ψ* with change in *α*: *α* = 0.8.

**Fig 9:**
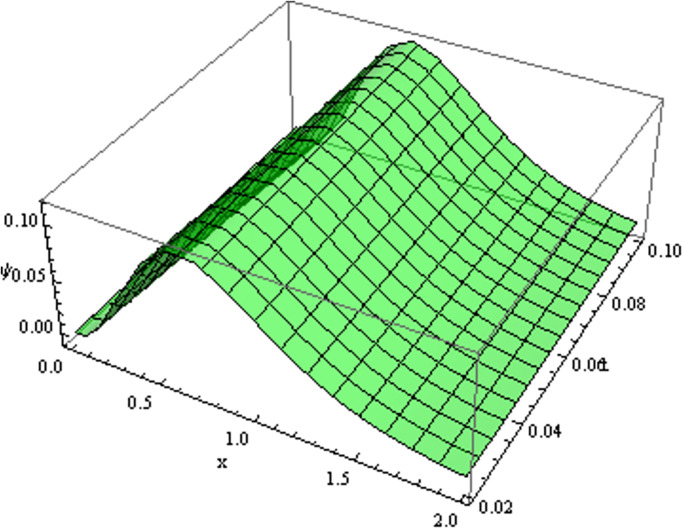
Variation of *ψ* with change in *α*: *α* = 1.

**Fig 10:**
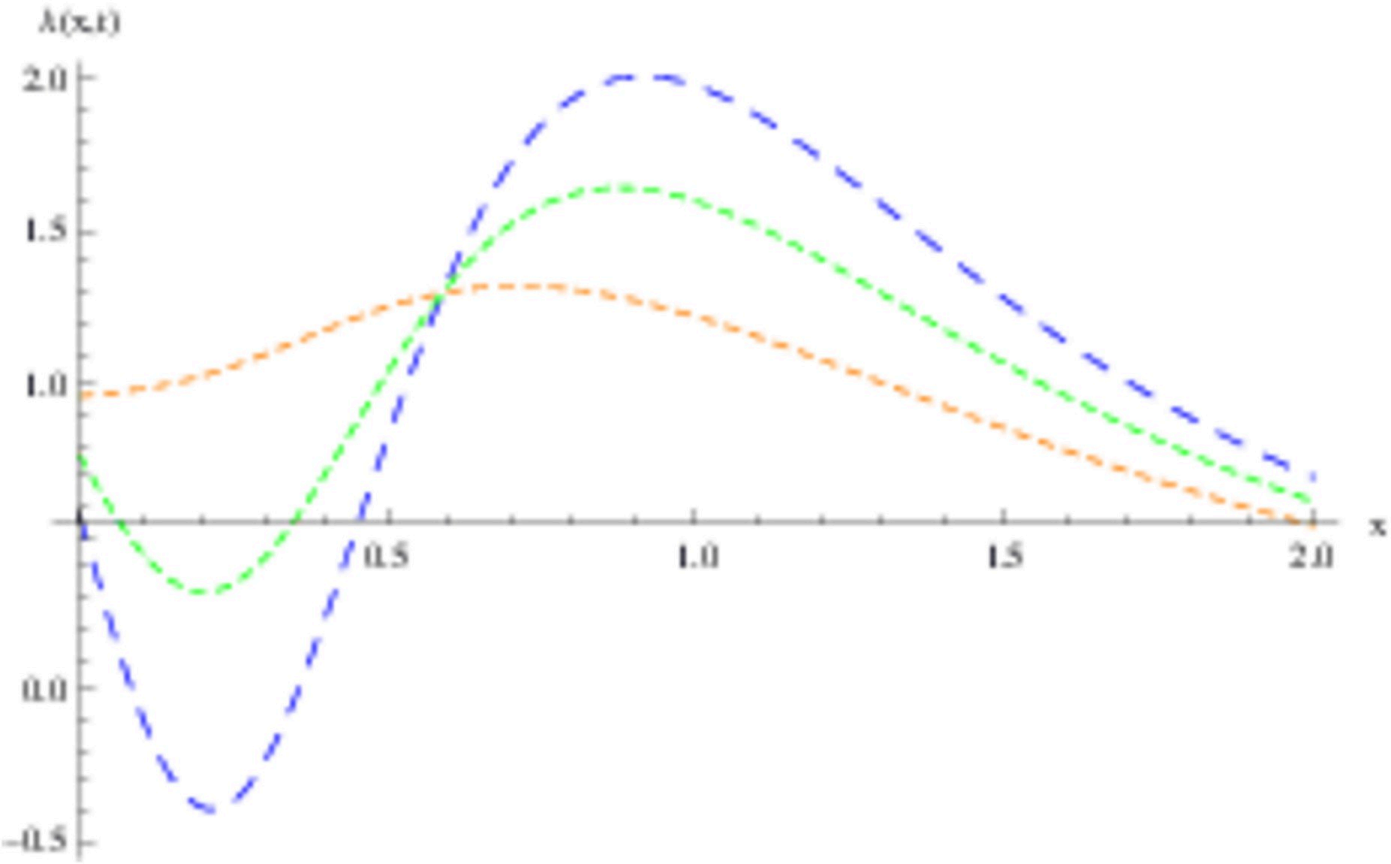
2D graph of λ with *α* = 0.6, *α* = 0.8 and *α* = 1: *t* = 0.04.

**Fig 11:**
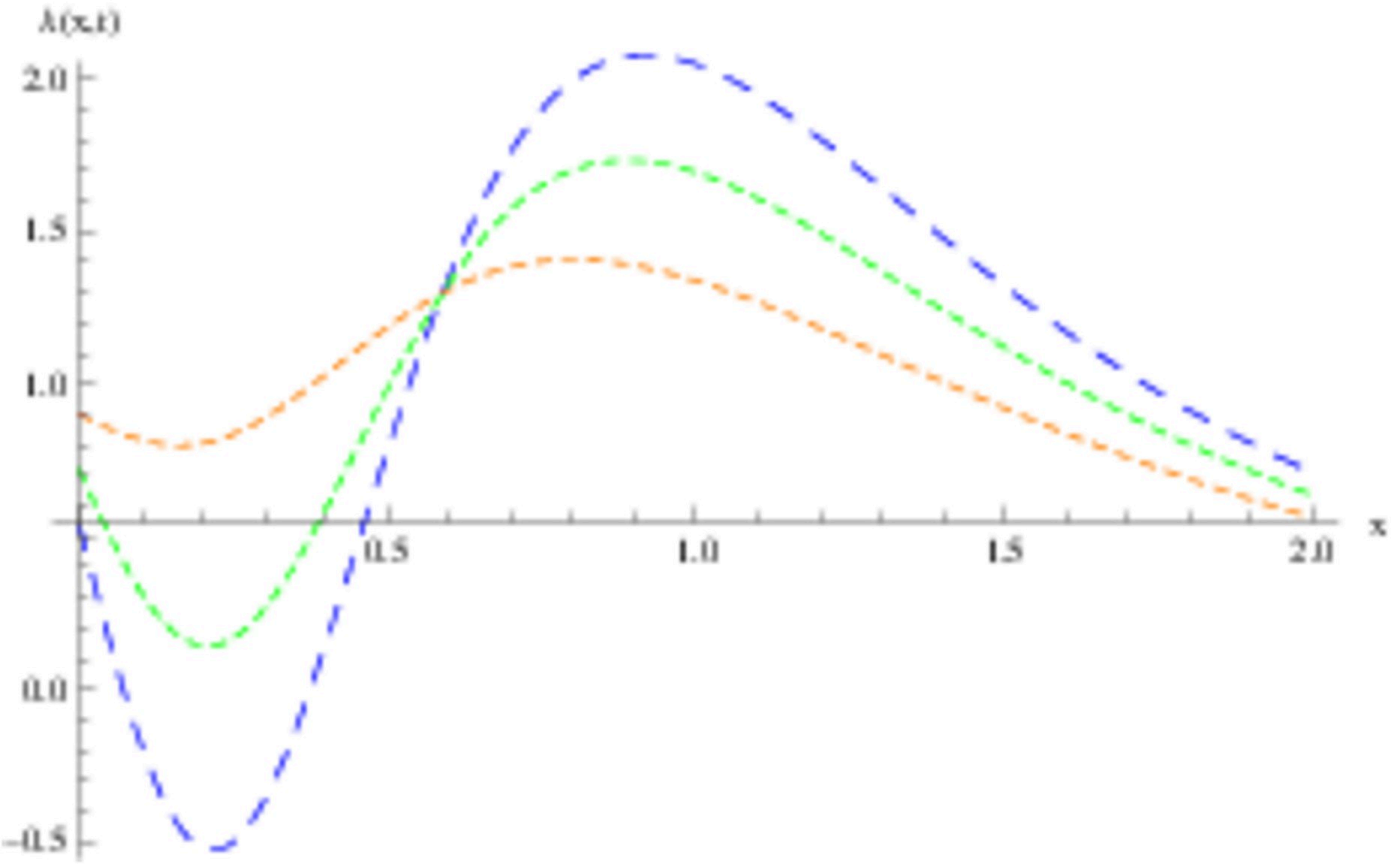
2D graph of λ with *α* = 0.6, *α* = 0.8 and *α* = 1: *t* = 0.1.

**Fig 12:**
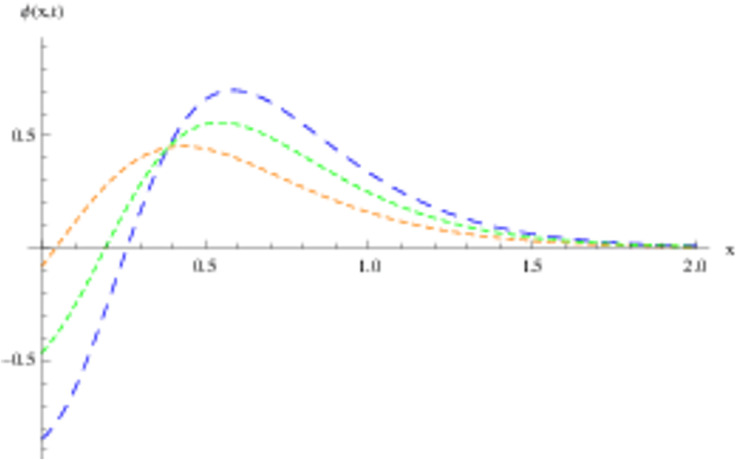
2D graph of *ϕ* with *α* = 0.6, *α* = 0.8 and *α* = 1: *t* = 0.04.

**Fig 13:**
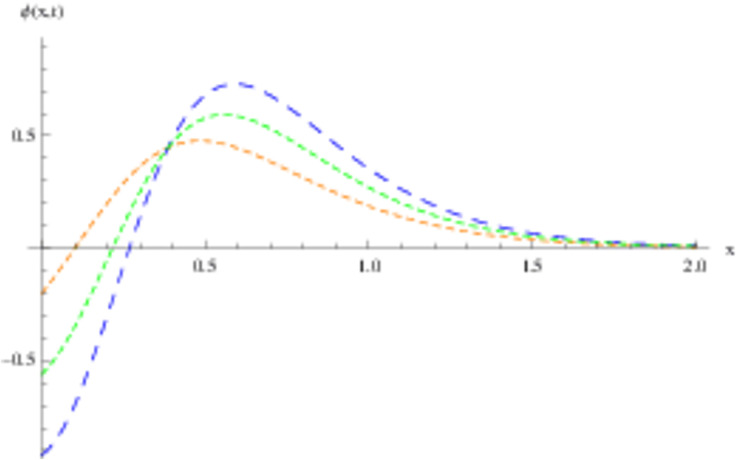
2D graph of *ϕ* with *α* = 0.6, *α* = 0.8 and *α* = 1: *t* = 0.1.

**Fig 14:**
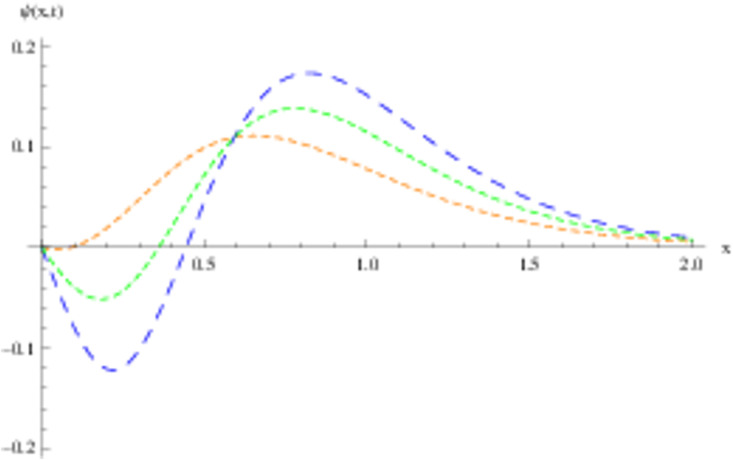
2D graph of *ψ* with *α* = 0.6, *α* = 0.8 and *α* = 1: *t* = 0.04.

**Fig 15:**
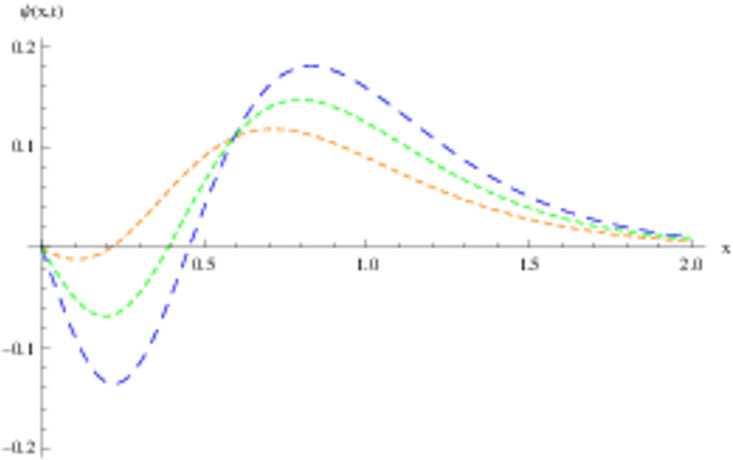
2D graph of *ψ* with *α* = 0.6, *α* = 0.8 and *α* = 1: *t* = 0.1.

The retrieved solutions are also explained with graphs presented in Figs 1–15. Figs 1–3 depict the change in the 3-dimensional plot of the solution function λ for increase in fractional parameter *α*. Figs 4–6 show the change in the 3-dimensional plot of the solution function *ϕ* for increase in fractional parameter *α*. Figs 7–9 represent the change in the 3-dimensional plot of the solution function *ψ* for increase in fractional parameter *α*. The colored lines in the 2-dimensional graphs depict the plot of functions at different values of *α*. The blue, green and orange lines correspond to *α* = 0.6, 0.8 and 1, respectively. Figs 10 and 11 show how the solution function λ is modified with increase in *α* for *t* = 0.04 and *t* = 0.1. Similar observations are presented for *ϕ* and *ψ* in Figs 12–15 respectively.

## 6 Results and discussion

The primary purpose of this work is to obtain the solution of a mathematical model for the atmospheric internal waves which is expressed as a set of three partial differential equations involving three unknown functions as given by (25). In the considered description, the CF fractional time derivative is used. The motivation for the use of CF derivative is the successful use of fractional derivatives in many recent works due to its generalized nature ad many interesting mathematical properties. The three unknown functions λ(*x*, *t*), *ϕ*(*x*, *t*) and *ψ*(*x*, *t*) are determined subject to given initial conditions by using the Elzaki Adomian decomposition method which are presented in Eqs (52)–(54).

Some numerical and graphical observations are presented for the obtained results. These observations depict several useful aspects of this study. Firstly, the solution of the fractional order mathematical model for the atmospheric internal waves is determined whose accuracy has been established by comparison with the previous literature. Secondly, the Elzaki Adomian decomposition method is applied for the first time to study this problem in this work which shows the reliability of the proposed method to deal with similar mathematical models. Thirdly, the it is observed in Section 5, that the graphs for the functions λ(*x*, *t*), *ϕ*(*x*, *t*) and *ψ*(*x*, *t*) continuously change with increasing value of the order of CF time derivative. The values of λ(*x*, *t*), *ϕ*(*x*, *t*) and *ψ*(*x*, *t*) with CF time derivative of order 1 ultimately coincide with the function values obtained for the integer order model in previous literature, thus confirming that considered CF model is a generalization of corresponding integer order model. It is hoped that these outcomes will be beneficial in future studies related to the presented work.

## 7 Conclusion

In this work, a time-fractional model for internal wave phenomenon in atmosphere is examined with CF fractional temporal operator. The considered time-fractional model is more generalized and flexible due to the memory and hereditary properties of time-fractional operator. The solution to the considered model is constructed by using the Elzaki Adomian decomposition method considering suitable initial conditions. The numerical results are graphically illustrated for different parametric values to understand the effect of variation in fractional order of derivative. The Elzaki Adomian decomposition method is advantageous to study the fractional atmospheric waves model due to its applicability and accuracy as observed in this work. Hence, this work underlines the importance and efficiency of the applied method. This method can also be used to analyze more nonlinear fractional differential equations in mathematical physics. The reported results are significant contribution for future studies as they may be useful in understanding and suggesting new experiments related to the propagation of internal waves in the atmosphere. In future, the stability and uniqueness of the proposed results for fractional order system will be investigated.
